# Development of a Novel, Sensitive, Selective, and Fast Methodology to Determine Malondialdehyde in Leaves of Melon Plants by Ultra-High-Performance Liquid Chromatography-Tandem Mass Spectrometry

**DOI:** 10.1155/2017/4327954

**Published:** 2017-01-19

**Authors:** Melisa E. Yonny, Ariel Rodríguez Torressi, Mónica A. Nazareno, Soledad Cerutti

**Affiliations:** ^1^CITSE-CONICET, Universidad Nacional de Santiago del Estero, 4200 Santiago del Estero, Argentina; ^2^INTA, 4200 Santiago del Estero, Argentina; ^3^Instituto de Química de San Luis (CONICET-UNSL), Área de Química Analítica, Facultad de Química, Bioquímica y Farmacia, Universidad Nacional de San Luis, 5700 San Luis, Argentina

## Abstract

Early production of melon plant* (Cucumis melo)* is carried out using tunnels structures, where extreme temperatures lead to high reactive oxygen species production and, hence, oxidative stress. Malondialdehyde (MDA) is a recognized biomarker of the advanced oxidative status in a biological system. Thus a reliable, sensitive, simple, selective, and rapid separative strategy based on ultra-high-performance liquid chromatography coupled to positive electrospray-tandem mass spectrometry (UPLC-(+)ESI-MS/MS) was developed for the first time to measure MDA, without derivatization, in leaves of melon plants exposed to stress conditions. The detection and quantitation limits were 0.02 *μ*g·L^−1^ and 0.08 *μ*g·L^−1^, respectively, which was demonstrated to be better than the methodologies currently reported in the literature. The accuracy values were between 96% and 104%. The precision intraday and interday values were 2.7% and 3.8%, respectively. The optimized methodology was applied to monitoring of changes in MDA levels between control and exposed to thermal stress conditions melon leaves samples. Important preliminary conclusions were obtained. Besides, a comparison between MDA levels in melon leaves quantified by the proposed method and the traditional thiobarbituric acid reactive species (TBARS) approach was undertaken. The MDA determination by TBARS could lead to unrealistic conclusions regarding the oxidative stress status in plants.

## 1. Introduction

Harsh environmental conditions such as drought, salinity, and extreme temperatures can delay growth and development of plants, inflict lethal injuries to the plant structure, and reduce crop yield. These abiotic stress factors cause the overproduction and accumulation of reactive oxygen species (ROS) [1]. ROS include free radicals like superoxide and peroxyl radicals as well as nonradical species such as singlet oxygen and hydrogen peroxide. Under abiotic stress condition, limitation of CO_2_ uptake, caused by stress-induced stomatal closure, favors photorespiratory production of H_2_O_2_ in the peroxisome and production of superoxide and H_2_O_2_ or singlet oxygen by the overreduced photosynthetic electron transport chain [2]. In this conditions, when the ROS production exceeds the intrinsic antioxidant defense mechanisms in a biological system, oxidative stress is produced causing damage to cell molecules and, hence, affecting the normal cell functions and ultimately cell death [3]. Thus it is interesting to note that the mentioned abiotic stress types lead to a high ROS production in the plant physiology and thus the oxidative stress [4]. In this sense, reductions of more than 50% in the plant growth for most major crop plants have been reported when they were exposed to abiotic and oxidative stress conditions [5]. These stresses produce plants damage in many ways: plant growth, membrane integrity, pigment content, osmotic adjustments, water relations, and photosynthetic activity [6, 7]. Research regarding how abiotic stresses affect plant growth and development at the physiological, biochemical, and molecular levels is critical to increasing the productivity of crops [8].

Melon plant* (Cucumis melo)* is a crop of high seasonality. However, in areas with temperate conditions during winter, early fruit production is possible and better market prices are obtained. For this purpose, melon crop is carried out using micro and macro structured tunnels covered by different types of materials, especially plastics. Under these conditions, extreme temperatures take place inducing abiotic stress in plants [9]. Temperature is one of the main environmental factors that affect plant growth and development since it leads to high ROS production and, hence, oxidative stress [9]. The primary targets of ROS are unsaturated lipids, components of cell membranes, leading to lipid oxidation reaction [3]. Among secondary lipid oxidation products, malondialdehyde (MDA) is one of the most recognized biomarkers of the advanced oxidative status in biological systems [10].

Several strategies have been carried out to measure MDA in a variety of biological samples such as plasma and urine [11], semen [12], and plants [13].

Most of the methodologies proposed to quantify MDA require a tedious derivatization step. Thus analytical approaches using hydrazine-based derivatization reagents have been reported. These strategies were coupled to separation techniques such as liquid or gas chromatography employing a wide variety of detection systems, such as UV [14], tandem mass spectrometry (MS/MS) [15, 16], and fluorescence [17], among others. However, one of the most serious drawbacks related to the use of hydrazine is that the derivatives have to be extracted, dried, and reconstituted before analysis and these multiple time consuming steps reduce its applicability to a large amount of samples.

Another interesting derivatization strategy to determine MDA concentrations is the condensation reaction with thiobarbituric acid (TBA), to give a colored product which can be spectrophotometrically measured at 532 nm or by fluorescence detection with excitation and emission at 530 nm and 550 nm, respectively [18]. This colorimetric assay is also known as the reactive species to TBA (TBARS) method. However, other molecules (saturated and unsaturated aldehydes) can also react with TBA under the same experimental conditions. Therefore, a lack of specificity towards MDA and overestimation of its levels due to the formation of additional light-absorbing and fluorescent species that contribute to the absorption in the same region of MDA-TBA_2_ condensation product is this method's main drawback [19]. To overcome these problems various analytical approaches have been proposed, especially mostly based on the resolution of the MDA-TBA_2_ condensation product using high performance liquid chromatography (HPLC) coupled to UV-Vis [20], fluorescence [19], and mass spectrometry (MS) detection [21], as well as capillary electrophoresis (CE) with UV-Vis detection [22]. Moreover, despite the mentioned limitations of the TBARS method, particularly its lack of specificity, it still remains to be reported as the main method to determine the presence of MDA in biological systems [23], probably due to the easy access to the necessary instrumentation.

On the other hand, several possibilities to determine MDA concentrations without using any derivatization procedure have been developed and reported. These methodologies used HPLC-UV [24], HPLC-MS/MS [25], and UHPLC-PDA [26] separation/detection systems.

Based on the above mentioned, the aim of this work was to develop and optimize a novel, selective, sensitive, and fast methodology to determine MDA in leaves of melon plants using ultra-high-performance liquid chromatography coupled to positive electrospray-tandem mass spectrometry (UPLC-(+)ESI-MS/MS), without using any derivatization reagent. To the best of our knowledge, the present work constitutes the first report related to the quantitative analysis of MDA levels in vegetal tissues by UPLC-(+)ESI-MS/MS. Particularly, herein, the MDA concentration was evaluated in leaves of melon plants to measure its oxidative status after being exposed to low temperatures as stress conditions. Thus this methodology allows the determination of ultratrace MDA concentrations in plants and makes it possible to find subtle and significant differences in MDA levels when plants are exposed to thermal stress. The detection and quantitation limits reached were comparable and inclusive better than the ones reported for others methodologies based on mass spectrometry detection [15, 16, 21, 25], as well as the methodologies that used the same matrix [13] or those that quantified MDA without derivatization [24, 26].

On the other hand, a comparison between the proposed methodology and the traditional TBARS method was carried out.

## 2. Experimental Section

### 2.1. Materials

As precursor of MDA, 1,1,3,3-tetraethoxypropane (TEP) was used. The TEP standard was purchased from Sigma (St. Louis, USA). Thiobarbituric acid (TBA) was provided from Merck (Darmstadt, Germany). Trichloroacetic acid (TCA) was acquired from Biopack (Buenos Aires, Argentina). Water, methanol, and acetonitrile of Optima® LC–MS grade were purchased from Fisher Scientific (Fair Lawn, NJ, USA). Formic acid (98%) was obtained from Fisher Scientific (Loughborough, UK). Reverse phase cartridges of 3 mL and 60 mg of Oasis HLB® were purchased from Waters (Milford, USA). Seeds of melon plants of the cv. Sweet Ball were provided from Rijkzwaan (Buenos Aires, Argentina). The seeds were sown in pots of 10 L filled with soil and compost in ratio 75/25 (v/v).

### 2.2. Stock and Working Solutions

A 10 mM TEP stock solution in 50 : 50 (v/v) methanol-water was prepared. Standard solutions, obtained by appropriate dilutions of the above-mentioned stock solution, containing 0.1% (v/v) formic acid were incubated for 30 min at 40°C to quantitatively release MDA from TEP and to use for calibration purposes.

### 2.3. Sample Preparation

Fresh leaves portion of melon plants (5 g) was used in validation and application studies. These samples were previously obtained from plants under thermal stress conditions. The samples were divided into two groups: stressed and nonstressed or control samples. The plants, when having 5 to 6 true leaves, were artificially treated in environments with low temperatures. Through these treatments the environmental conditions were simulated that normally affect plants of recent transplant plants in the early production melon systems at the end of the winter season. Cold treatments were performed introducing the plants into a cooling camera with temperature control. Specifically, stressed samples were exposed to temperatures between 0°C and 4°C, while control samples were kept at room temperature (25–30°C). MDA extraction of plant tissues was carried out as described by Djanaguiraman et al. [27], with some changes. Leaves were processed with a tissue homogenizer Bio-Gen PRO200 at 17000 rpm, using 10 mL of 0.1% (v/v) formic acid as extraction solvent. Afterwards, the extract was centrifuged at 15000 g (11500 rpm) at 4°C for 15 min. MDA levels were determined by different methods as follows.

### 2.4. TBARS Method

A 1 mL aliquot of supernatant extract was mixed with 4 mL of 0.5% (w/v) TBA in 20% (w/v) TCA. The reaction mixture was heated at 95°C for 30 min. Then, it was cooled in ice bath for 10 min and was centrifuged at 10000 g at 4°C for 10 min. The obtained supernatant was separated and analyzed by spectrophotometry for MDA levels determination [28].

### 2.5. UPLC-MS/MS Method

On the other hand, another portion of 2.0 mL of each supernatant extract was filtered through a 0.2 *μ*m polytetrafluoroethylene syringe filter into a glass vial prior to underivatized MDA analysis by UPLC-MS/MS. In this case, to reduce the observed matrix effect (90% suppression) on the MDA analytical response, a clean-up strategy through a solid-phase extraction (SPE) procedure was performed. For SPE, Oasis HLB reverse phase cartridges were used. Aliquots of 2 mL sample were applied to the cartridges previously conditioned with 2 mL of methanol and equilibrated with 2 mL of 5% (v/v) methanol. The retained fraction was eluted with 2 mL of 10% (v/v) acetonitrile containing 0.1% (v/v) formic acid. Following this SPE procedure, the MDA recovery was above 90% and the matrix effect decreased up to a 10% after SPE clean-up.

### 2.6. Experimental Conditions for TBARS Analysis

Spectrophotometric analyses of TBARS were carried out in a Unicam UV2 equipment. Absorption spectra were scanned between 200 and 700 nm and absorbance was measured at 532 nm.

### 2.7. Liquid Chromatography and Mass Spectrometry Conditions

An Acquity® ultra-high-performance liquid chromatography system (Waters, Milford) equipped with autosampler injection and pump systems (Waters, Milford) was used. The autosampler vial tray was kept at 15°C. The needle was washed with proper mixtures of acetonitrile and water. The separation was performed by injecting a 10 *μ*L sample volume onto an ACQUITY UPLC® BEH C_18_ (Waters, Milford, USA) analytical column with 2.1 mm internal diameter, 50 mm length, and 1.7 *μ*m particle size. The binary mobile phases consisted of water with 0.1% (v/v) of formic acid (A) and acetonitrile with 0.1% (v/v) of formic acid (B) delivered at 0.1 mL min^−1^. The elution was isocratic with a mobile phase composition of 70% A and 30% B. The total run time, including conditioning time, was 3.0 min. The column was held at a temperature of 25°C. Under these conditions, no sample contamination or sample to sample carryover was observed.

Mass spectrometry analyses were performed on a Quattro Premier® XE Micromass MS Technologies triple quadrupole mass spectrometer with a Z-Spray® electrospray ionization source (Waters, Milford, USA). The source was operated in the positive (ESI+) mode at 300°C with N_2_ as the nebulizer and the source temperature was kept at 150°C. The capillary voltage was maintained at 1.9 kV and the extractor voltage was set at 4.0 kV. Ultrapure nitrogen was used as desolvation gas with a flow of 850 L h^−1^.

Detection of the MDA protonated molecule [M + H]^+^ of* m*/*z* 73 was performed in the selected ion monitoring mode (SIR). To choose the maximum sensitivity conditions, direct infusion (via syringe pump) into the MS of MDA standard solution in 0.1% (v/v) formic acid was performed and the product ion scan mass spectra were recorded over a* m*/*z* range of 50–150. Quantification of MDA was done by measuring the area under the specific peak using MassLynx Mass Spectrometry Software (Waters, Milford, USA).

### 2.8. Assay Validation

The calibration plots were measured under the optimal experimental conditions. Six levels of the calibration curve were assayed: 50, 100, 200, 500, 1000, and 1500 *μ*g·L^−1^, with three technical replicates at each concentration level. The calibration equations were calculated by the least-squares linear regression method.

In order to estimate the trueness, intraday repeatability, and interday reproducibility, homogenous samples with the addition of MDA standard were analyzed. Relative standard deviation (RSD) was used as a measure of precision. The intraday precision was assessed by making repetitive injections of spiked leaves extracts (*n* = 6) samples under the selected optimum conditions. The interday precision was estimated by making repetitive injections of spiked leaves extracts samples during three consecutive days.

To further evaluate the accuracy of the method, recovery experiments were performed by the addition of known amounts of standard MDA to a homogeneous sample (prior to SPE and UPLC-MS/MS analysis), to achieve different concentrations within linear range.

Sensitivity of the method was determined in terms of limit of detection (LOD) and limit of quantitation (LOQ). These limits were calculated taking into account the ratios of 3.3 and 10 times between the standard deviation of the blank response and the slope of the calibration curve [29].

### 2.9. Statistical Analyses

Analysis of variance (ANOVA) was used to determine significant differences among data. Each statistical analysis was done using the software program INFOSTAT version 2012 (Universidad Nacional de Cordoba). Fisher LSD-test was used to compare means when the effects were found to be significant (*P* < 0.05).

## 3. Results and Discussion

### 3.1. Optimization of UPLC and MS/MS Conditions

Gradient and isocratic elution conditions were evaluated and the results showed that isocratic elution using a mixture of water/ACN in a 70 : 30 proportion was optimum for MDA release from the column. This result is in agreement with Syslová et al. [25], who found that MDA elution occurs in an isocratic section of the chromatographic run and when the proportion of aqueous component of the mobile phase is higher than the organic component. To enhance the signal response in the MS/MS system, mobile phase modifiers such as acetic acid and formic acid, at different concentrations, were also studied. The results observed with formic acid led to an improved peak shape and shorter retention time than those obtained with acetic acid. Consequently, a 0.1% (v/v) formic acid concentration was used as a mobile phase modifier to provide the maximum response for the generation of the MDA protonated molecule [M + H]^+^.

**Figure 1:**
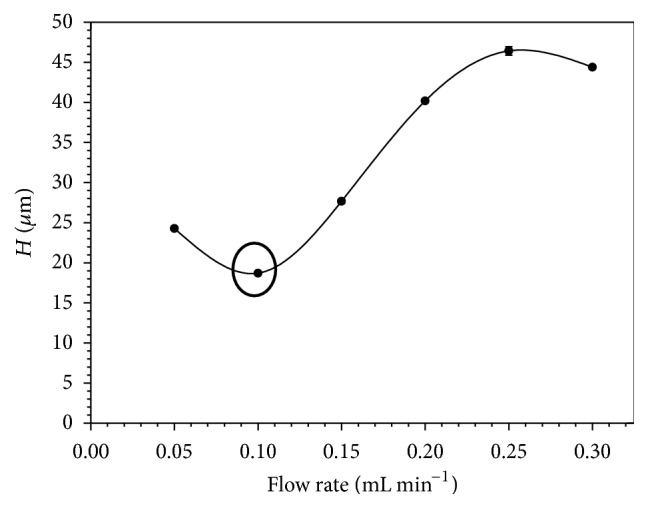
Van Deemter curve for MDA. The conditions were as follows: UPLC C_18_ column; isocratic elution, mobile phase containing acetonitrile-water with 0.1% (v/v) formic acid; temperature 25°C; MDA concentration 100 *μ*g L^−1^; injection volume 10 *μ*L.

**Figure 2:**
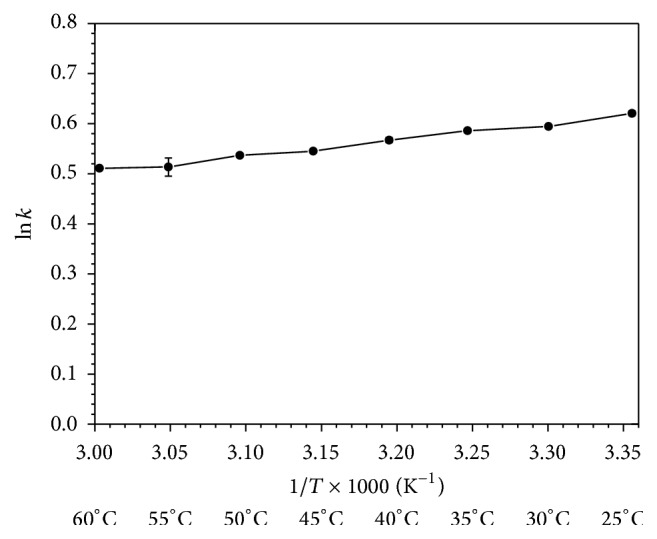
Van't Hoff plot for MDA. The conditions were as follows: UPLC C_18_ column; isocratic elution, mobile phase containing acetonitrile-water with 0.1% (v/v) formic acid; flow rate 0.1 mL min^−1^; MDA concentration 100 *μ*g L^−1^; injection volume 10 *μ*L.

**Figure 3:**
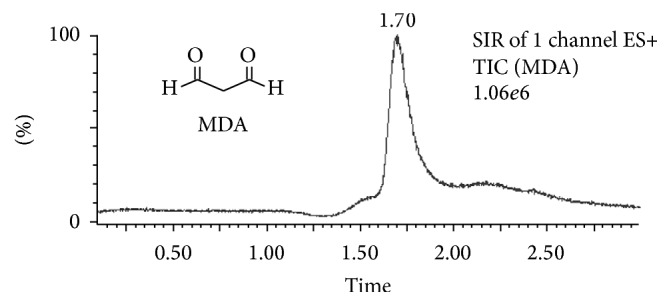
MDA chromatogram obtained with a UPLC C_18_ column associated with (+)ESI-MS/MS after sample SPE treatment. The analyte was quantified using the selected ion monitoring (SIR) mode.

The effect of the mobile phase flow rate on the separation/retention MDA was evaluated using van Deemter plots. A 10 *μ*L standard sample injection volume was loaded onto the system at several flow rates, from 0.05 to 0.3 mL min^−1^. A flow rate of 0.1 mL min^−1^ showed the best results in terms of chromatographic conditions and ESI efficiency (Figure 1). In addition, the effect of column temperature on the retention of MDA was studied. The Van't Hoff plot in a temperature interval from 25 to 60°C was evaluated. The elution time of MDA decreased as the column temperature increased. The optimal retention conditions were obtained when the temperature was fixed at 25°C (Figure 2). This temperature was selected for further experiments. Under these optimal conditions, MDA was eluted from the column at 1.70 min in a total run cycle of 3.00 min, as shown in Figure 3.

In previous reports Moselhy et al. [21] and Syslová et al. [25] reported the MDA quantification being performed using ESI, but configured in a negative polarity mode, as strategy for ionization, with and without MDA derivatization. In the herein presented work, after the ESI source parameters were optimized (polarity, capillary voltage, source temperature, probe temperature, drying gas flow rate, and drying gas temperature) the proper values were achieved, as mentioned in the “mass spectrometry conditions” section, but being the ESI source in positive mode is the best strategy for MDA ionization. The analytical response for the generation of the MDA protonated molecule [M + H]^+^ was higher than the obtained for the generated [M − H]^−^ ion when the negative ESI mode was assayed.

Preliminary experiments were conducted to find the best instrumental conditions that allow the analysis of underivatized MDA in leaves samples. A MDA standard solution (1.0 mg L^−1^) in 0.1% (v/v) formic acid was introduced into the MS system at a flow rate of 50 *μ*L min^−1^ via a syringe pump. The positive ion full scan (from* m*/*z* 50 to* m*/*z* 150) indicated the presence of the MDA protonated molecule, [M + H]^+^, as the predominant specie, with a* m*/*z* value of 73.1. The product ion mass spectra in Multiple Reaction Monitoring (MRM) mode were assayed. As a result, the specific charged fragments obtained by collision-induced dissociation (CID) of protonated molecule previously isolated were 45.4 and 55.4. Despite optimization, the intensities observed for these fragments were not enough and compatible with the quantitation purposes of this work. Then, the detection in Selected Ion Register (SIR) mode was chosen as the best alternative for MDA evaluation in melon leaves. Additionally, the atmospheric-pressure chemical ionization source operating in positive or negative mode (APCI) was also assayed. The results showed that the ESI source allowed to obtain an ionization efficiency at least an order of magnitude higher than the APCI source.

### 3.2. Sample Clean-Up by SPE

In the ESI-MS or ESI-MS/MS configurations, the ionization process is susceptible to signal suppression or enhancement [30]. For this, ion suppression combined with SPE performance on MDA signal was evaluated.

Initially, it was found that the sample matrix produced approximately 90–100% of signal suppression in the MDA determination when the melon leaves extracts, with MDA standard added, were analyzed directly by UPLC-(+)ESI-MS/MS proposed method. To minimize this, SPE reverse phase cartridges were used. Samples spiked with MDA standard at different concentrations and treated with SPE were compared with samples spiked without SPE. An improvement in the MDA signal detection was achieved, making the overall suppression effect decrease from 90–100% to approximately 10%. Then, the calibration curve was created with the homogeneous leaf samples spiked (standard addition method with 50, 100, 300, 500, and 1000 *μ*g·L^−1^ MDA addition levels) and with SPE treatment. The ratio between calibration slopes in pure solvent (formic acid 0.1%) and in sample matrix was calculated as the index of signal suppression degree. Finally, an 8.96% of signal suppression was registered.

### 3.3. Method Validation

**Figure 4:**
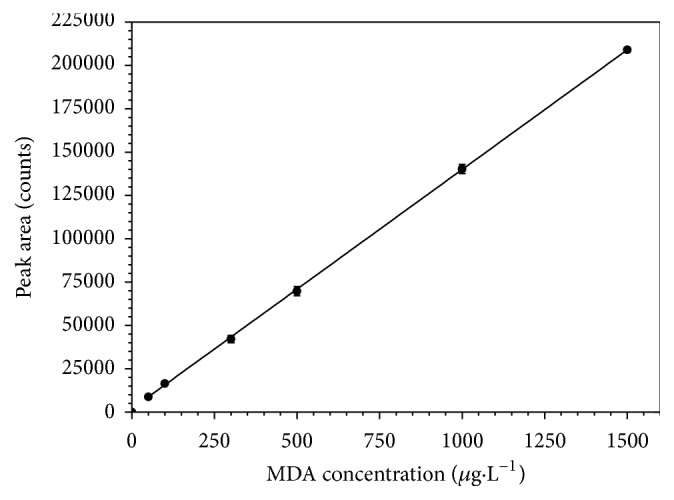
MDA calibration curve. MDA concentrations tested between 10 and 1500 *μ*g·L^−1^. Regression line: *y* = 2400 + 134*x*, with *R*^2^ = 0.9996.

As shown in Figure 4, an excellent linearity between peak area and analyte concentration could be obtained in a range of at least two orders of magnitude. The LOD and LOQ values were 0.02 and 0.08 *μ*g·L^−1^, respectively. These values were comparable and in some cases better than the ones reported for others methodologies based on liquid chromatography with mass spectrometry and UV detection, with or without MDA derivatization (Table 1).

On the other hand, intraday and interday precisions, expressed as relative standard deviations (% RSD), were 2.7% and 3.8%, respectively (Table 2). Recovery studies demonstrated recoveries of 104, 98, 97, and 96% for 200, 300, 500, and 1000 *μ*g·L^−1^ MDA spiked samples, respectively (Table 2). The above-mentioned results indicated the feasibility to determine the MDA concentration by the proposed methodology without needing a derivatization agent.

In addition to the figures of merit mentioned above, 20 samples per hour can be analyzed with the proposed method and the results could be translated into an improvement in the crop production planning.

In summary, taking into account the matrix complexity, the reported values for the validation parameters corresponding to the proposed method can be considered highly satisfactory.

### 3.4. Application Assay

The optimized methodology was used to evaluate the MDA concentration in fresh leaves of melon plants. As mentioned earlier, these plants were raised under two different conditions: normal (control samples) and thermal stress conditions (stressed samples). As shown in Table 3, average MDA concentration levels of control and stressed samples were statistically different (*P* = 0.0027). Therefore, the proposed method allowed not only the quantification of MDA levels in the studied samples, but also the detection of subtle significant changes in the levels of the target compound in plants grown under different stress conditions. These preliminary results open a great number of research opportunities in order to evaluate the plant resistance (and productivity) to thermal stress and thus to reduce its effects.

### 3.5. Comparison with MDA Levels by TBARS Method

A comparison between the MDA levels obtained from this study by the UPLC-MS/MS proposed method and the MDA levels by the traditional TBARS method was undertaken. (Table 3). Quantification of MDA by spectrophotometric analysis resulted in the fact that MDA concentrations for both the control and the stressed samples were not statistically different (*P* = 0.2087) and were up to three times higher than the MDA concentrations determined by the proposed UPLC-MS/MS methodology. This TBARS overestimation in MDA levels was more pronounced in the case of control samples. This could be explained taking into account that, in the situation of the control samples, the MDA level product of basal lipid oxidation can be smaller compared to the nonspecific background reaction between TBA and products not derived from lipid oxidation [31]. Thus, the effect of interferences in the control samples is greater than in the stressed samples. Besides, the results of the present work are in agreement with those reported by Tug et al. [32], who found that the MDA concentrations determined by the traditional TBARS method in the control samples were five times higher than the ones quantified by HPLC and in stressed samples the MDA concentrations determined by TBARS method were three times higher than the ones quantified by HPLC. Although the differences between the MDA levels determined by both the TBARS and the UPLC-MS/MS method, decreased when the MDA levels increased, the overestimation of the MDA concentrations, mainly in the control samples, by the TBARS method argues its reliability and suitability for application to MDA quantification of true differences in the lipid peroxidation levels [32].

In this sense, a methodology to quantify MDA with TBARS assay and overcome the interferences has been proposed [33]. In this methodology 2nd derivative analysis of the absorption spectra was applied and if well a MDA level quantified was approximately 1.6- and 2.1-fold lower than with the TBARS traditional assay, the sensitivity reached is only 0.2 *μ*M (1.4 × 10^7^ *μ*g·L^−1^). Taking into account this sensitivity value, subtle differences in MDA content could be not found.

Finally the importance of the proposed UPLC-MS/MS method is that it allowed finding reliable differences between control and stressed samples, and these changes were not found with the TBARS strategy as a consequence of the MDA overestimation in the control samples. In this sense, a conclusion from the obtained results with the TBARS method could be that the thermal stress does not induce an oxidative stress in the plant system. However, the reliable results obtained with the MDA determination using the proposed separative methodology allowed relating thermal stress with oxidative stress because the MDA levels in the plants exposed to low temperatures (stressed plants) were higher than those found in the control plants. This situation clearly demonstrates that the TBARS method could induce wrong results.

## 4. Conclusion

An UPLC-MS/MS method has been developed to determine MDA in vegetal tissue, particularly as a stress biomarker in fresh leaves of melon plants. From an analytical perspective, methodologies of MDA analysis in plant samples by LC- (or UPLC-) MS/MS have not been reported. Thus the methodology developed in the present work has proved to be simple, rapid, reliable, sensitive, selective, and reproducible for the determination of MDA and its variations, without needing a derivatization procedure. The proposed method allowed the quantification of levels of MDA as an oxidative stress marker in leaves of melon plants grown under thermal stress conditions and provided a valuable tool for MDA routine analysis as a biomarker of the oxidative stress and of the metabolic activity of the plant index.

In addition, the comparison between the MDA levels determined by UPLC-MS/MS and by the traditional TBARS method demonstrated the unspecific response and overestimation of the photometric approach and its inadequacy to discriminate significant differences in the sample oxidative status when the samples were in different growth conditions. This overestimation of the MDA concentration levels using the TBARS methodology could lead to unrealistic conclusions regarding the plant oxidative status.

The interferences in the MDA determination were eliminated using the proposed UPLC-MS/MS methodology instead of the TBARS method. The advantage of the separative method is that statistical differences could be found among MDA levels corresponding to the different plant stress status. Thus, the MDA quantitation as biomarker of the oxidation advance using the UPLC-MS/MS methodology constitutes a viable tool to evaluate the oxidative status in melon plants exposed to different conditions. Moreover, knowledge of feasible antioxidants therapies that can be applied to reduce the plant oxidative stress could be obtained.

## Figures and Tables

**Table 1:** Comparison of the analytical performance of the proposed methodology with others previously reported in the literature.

Detection technique	Separation technology	Derivatization reagent (derivatization time)	Matrix	Sample clean-up	LOD (*μ*g L^−1^)	LOQ (*μ*g L^−1^)	References
MS/MS	UPLC (BEH C_18_)	—	Plant tissue	SPE (OASIS HLB)	0.020	0.080	This study
MS/MS	HPLC (Hypercarb porous-graphite)	—	Urine	SPE	0.087	0.105	[25]
			Plasma	Protein precipitation with acetonitrile	0.031	0.039	
			Exhaled breath	SPE	0.021	0.032	
MS	HPLC (Hyperclone C_18_)	TBA (40 min)	Plasma	Hydrolysis with sodium hydroxide and protein precipitation with sulphuric acid	62	206	[21]
MS/MS	HPLC (C_18_)	4,2-trimethylammonio ethoxybenzenaminiumHalide or 4-APC (240 min)	Plasma	Protein precipitation with acetonitrile and on-line weak-cation exchange SPE (WCXE)	0.036	—	[16]
MS/MS	HPLC (C_18_)	2,4-dinitrophenylhydrazine or DNPH (70 min)	Urine	Automated SPE	0.11	0.46	[15]
PDA	UHPLC (HSS T3 strength silica)	—	Urine	Microextraction by packed sorbent (eVols-MEPS)	0.72	1.57	[26]
UV	HPLC (C_18_)	—	Serum	Protein precipitation with perchloric acid	0.86	—	[24]
PDA	Rocket HPLC (C_18_)	TBA (30 min)	Plant tissue	Protein precipitation with metaphosphoric acid	0.022	—	[13]

**Table 2:** Recovery study and analytical performance of the proposed SPE-UPLC-(+)ESI-MS/MS methodology for the MDA determination in melon leaves.

MDA added (*µ*g·L^−1^)	MDA found (*µ*g·L^−1^)			Precision (RSD %)				Accuracy (% recovery)		
	Day 1 (*n* = 6)	Day 2 (*n* = 6)	Day 3 (*n* = 6)	Day 1	Day 2	Day 3	Interday	Day 1	Day 2	Day 3
0	425 ± 11	414 ± 12	442 ± 11	2.6	2.9	2.5	3.3	—	—	—
200	631 ± 18	639 ± 19	680 ± 20	2.8	3.0	2.9	4.0	101	104	106
300	747 ± 21	707 ± 19	691 ± 17	2.8	2.7	2.5	4.0	103	99	93
500	869 ± 23	886 ± 24	942 ± 26	2.6	2.7	2.8	4.2	94	97	100
1000	1425 ± 35	1357 ± 33	1341 ± 33	2.5	2.4	2.5	3.7	100	96	93

Values are means ± standard deviation.

**Table 3:** Comparison between MDA levels determined by the proposed UPLC-MS/MS method and the traditional TBARS method.

Sample type	MDA level (*µ*g/g fresh weight)	
	UPLC-MS/MS	TBARS
Control	1.10 ± 0.03^^*∗*^a1^	3.6 ± 0.1^^*∗*^a2^
Stressed	3.5 ± 0.1^^*∗*^b1^	4.7 ± 0.2^^*∗*^a2^

^*∗*^a1 and b1 letters represent statistically different MDA concentration levels (*P* = 0.0027, *n* = 6), while the letter a2 represents average MDA values that were not statistically different (*P* = 0.2087, *n* = 6).

## Abbreviations

ACN: Acetonitrile
HPLC: High performance liquid chromatography
LOD: Limit of detection
LOQ: Limit of quantitation
MDA: Malondialdehyde
ROS: Reactive oxygen species
PDA: Photodiode array detection
RSD: Relative standard deviation
SIR: Selected ion monitoring mode
TBA: Thiobarbituric acid
TBARS: Thiobarbituric acid reactive species method
TCA: Trichloroacetic acid
TEP: 1,1,3,3-Tetraethoxypropane
UPLC-(+)ESI-MS/MS: Ultra-high-performance liquid chromatography coupled to positive electrospray-tandem mass spectrometry.

## References

[1] Demidchik V. (2015). Mechanisms of oxidative stress in plants: from classical chemistry to cell biology. *Environmental and Experimental Botany*.

[2] Noctor G., Mhamdi A., Foyer C. H. (2014). The roles of reactive oxygen metabolism in drought: not so cut and dried. *Plant Physiology*.

[3] Diplock A. T. (1998). Defence against reactive oxygen species. *Free Radical Research*.

[4] Hasanuzzaman M., Hossain M. A., Teixeira da Silva J. A., Fujita M., Bandi V., Shanker A. K., Shanker C., Mandapaka M. (2012). Plant responses and tolerance to abiotic oxidative stress: antioxidant defenses is a key factors. *Crop Stress and Its Management: Perspectives and Strategies*.

[5] Wang W., Vinocur B., Altman A. (2003). Plant responses to drought, salinity and extreme temperatures: towards genetic engineering for stress tolerance. *Planta*.

[6] Sanghera G. S., Wani S. H., Hussain W., Singh N. B. (2011). Engineering cold stress tolerance in crop plants. *Current Genomics*.

[7] Pathak M. R., da Silva Teixeira J. A., Wani S. H. (2014). Polyamines in response to abiotic stress tolerance through transgenic approaches. *GM Crops Food*.

[8] Kazan K. (2015). Diverse roles of jasmonates and ethylene in abiotic stress tolerance. *Trends in Plant Science*.

[9] Gülen H., Çetinkaya C., Kadıoğlu M., Kesici M., Cansev A., Eriş A. (2008). Peroxidase activity and lipid peroxidation in strawberry (Fragaria X ananassa) plants under low temperature. *Journal of Biological and Environmental Science*.

[10] Del Rio D., Stewart A. J., Pellegrini N. (2005). A review of recent studies on malondialdehyde as toxic molecule and biological marker of oxidative stress. *Nutrition, Metabolism and Cardiovascular Diseases*.

[11] Zhang G., Tang Y., Shi X. (2013). A chemiluminescence method to detect malondialdehyde in plasma and urine. *Analytical Biochemistry*.

[12] Yonny M. E., Reineri P. S., Palma G. A., Nazareno M. A. (2015). Development of an analytical method to determine malondialdehyde as an oxidative marker in cryopreserved bovine semen. *Analytical Methods*.

[13] Davey M. W., Stals E., Panis B., Keulemans J., Swennen R. L. (2005). High-throughput determination of malondialdehyde in plant tissues. *Analytical Biochemistry*.

[14] Korchazhkina O. (2003). Measurement by reverse-phase high performance liquid chromatography of malondialdehyde in normal human urine following derivatization with 2, 4-dinitrophenylhidrazine. *Journal of Chromatography B*.

[15] Chen J.-L., Huang Y.-J., Pan C.-H., Hu C.-W., Chao M.-R. (2011). Determination of urinary malondialdehyde by isotope dilution LC-MS/MS with automated solid-phase extraction: a cautionary note on derivatization optimization. *Free Radical Biology and Medicine*.

[16] Eggink M., Charret S., Wijtmans M. (2009). Development of an on-line weak-cation exchange liquid chromatography–tandem mass spectrometric method for screening aldehyde products in biological matrices. *Journal of Chromatography B*.

[17] Li P., Ding G., Deng Y., Punyapitak D., Li D., Cao Y. (2013). Determination of malondialdehyde in biological fluids by high-performance liquid chromatography using rhodamine B hydrazide as the derivatization reagent. *Free Radical Biology and Medicine*.

[18] Janero D. R. (1990). Malondialdehyde and thiobarbituric acid-reactivity as diagnostic indices of lipid peroxidation and peroxidative tissue injury. *Free Radical Biology and Medicine*.

[19] Lykkesfedelt J. (2001). Determination of malondialdehyde as thiobarbituric acid adduct in biological samples by HPLC with fluorescence detection: comparison with ultraviolet-visible spectrophotometry. *Clinical Chemistry*.

[20] Ying X., Li H., Xiong Z. (2008). LC determination of malondialdehyde concentrations in the human umbilical vein endothelial cell culture medium: application to the antioxidant effect of vitexin-2^″^-O-rhamnoside. *Chromatographia*.

[21] Moselhy H. F., Reid R. G., Yousef S., Boyle S. P. (2013). A specific, accurate, and sensitive measure of total plasma malondialdehyde by HPLC. *Journal of Lipid Research*.

[22] Cooley J. C., Lunte C. E. (2011). Detection of malondialdehyde in vivo using microdialysis sampling with CE-fluorescence. *Electrophoresis*.

[23] Dasgupta A., Dasgupta A., Klein K. (2014). Methods for measuring oxidative stress in the laboratory. *Antioxidants in Food, Vitamins and Supplements Prevention and Treatment of Disease*.

[24] Karatas F., Karatepe M., Baysar A. (2002). Determination of free malondialdehyde in human serum by high-performance liquid chroma tography. *Analytical Biochemistry*.

[25] Syslová K., Kačer P., Kuzma M. (2009). Rapid and easy method for monitoring oxidative stress markers in body fluids of patients with asbestos or silica-induced lung diseases. *Journal of Chromatography B*.

[26] Mendes B., Silva P., Mendonça I., Pereira J., Câmara J. (2013). A new and fast methodology to assess oxidative damage in cardiovascular diseases risk development through Vol-MEPS–UHPLC analysis of four urinary biomarkers. *Talanta*.

[27] Djanaguiraman M., Prasad P. V. V., Seppanen M. (2010). Selenium protects sorghum leaves from oxidative damage under high temperature stress by enhancing antioxidant defense system. *Plant Physiology and Biochemistry*.

[28] Kosugi H., Kato T., Kikugawa K. (1988). Formation of red pigment by a two-step 2-thiobarbituric acid reaction of alka-2,4-dienals. Potential products of lipid oxidation. *Lipids*.

[29] (1996). International Conference Harmonisation Topic Q2B, validation of analytical methods: methodology. *Pharmeuropa*.

[30] Constantopoulos T. L., Jackson G. S., Enke C. G. (1999). Effects of salt concentration on analyte response using electrospray ionization mass spectrometry. *Journal of the American Society for Mass Spectrometry*.

[31] Grotto D., Santa Maria L., Valentini J. (2009). Importance of the lipid peroxidation biomarkers and methodological aspects FOR malondialdehyde quantification. *Química Nova*.

[32] Tug T., Karatas F., Terzi S. M., Ozdemir N. (2005). Comparison of serum malondialdehyde levels determined by two different methods in patients with COPD: HPLC or TBARS methods. *Laboratory Medicine*.

[33] Merzlyak M. N., Zhigalova T. V., Shevyryova V. V. (1992). Assay of the thiobarbituric acid-reactive products of lipid peroxidation in plants using derivative absorption spectroscopy. *Phytochemical Analysis*.

